# The ‘PREXCEL-Q Method’ for qPCR

**Published:** 2008-12

**Authors:** Jack M. Gallup, Mark R. Ackermann

**Affiliations:** *Department of Veterinary Pathology, College of Veterinary Medicine, Iowa State University, Ames, Iowa, USA*

**Keywords:** PCR, qPCR, RT, gene expression, inhibition, RNA integrity, micro-array, real-time PCR, software

## Abstract

The purpose of this manuscript is to describe a reliable approach to quantitative real-time polymerase chain reaction (qPCR) assay development and project management, which is currently embodied in the Excel 2003-based software program named “PREXCEL-Q” (P-Q) (formerly known as “FocusField2-6Gallup-qPCRSet-upTool-001,” “FF2-6-001 qPCR set-up tool” or “Iowa State University Research Foundation [ISURF] project #03407”). Since its inception from 1997-2007, the program has been well-received and requested around the world and was recently unveiled by its inventor at the 2008 Cambridge Healthtech Institute’s Fourth Annual qPCR Conference in San Diego, CA. P-Q was subsequently mentioned in a review article by Stephen A. Bustin, an acknowledged leader in the qPCR field. Due to its success and growing popularity, and the fact that P-Q introduces a unique/defined approach to qPCR, a concise description of what the program is and what it does has become important. Sample-related inhibitory problems of the qPCR assay, sample concentration limitations, nuclease-treatment, reverse transcription (RT) and master mix formulations are all addressed by the program, enabling investigators to quickly, consistently and confidently design uninhibited, dynamically-sound, LOG-linear-amplification-capable, high-efficiency-of-amplification reactions for any type of qPCR. The current version of the program can handle an infinite number of samples.

## PREFACE

As real-time fluorogenic quantitative polymerase chain reaction (qPCR) is now accepted as the most powerful tool in all of molecular biology for quantitative analysis of gene expression, and since it is the tool of choice for validating gene micro-array and other data, any new implement that improves its execution represents an important constructive advance in furthering the responsible evolution of an important scientific technique (1). Despite its widespread use and essential role in most medical, biological, and life-science laboratories, qPCR is challenging from a technical standpoint due to: 1) the numerous calculations required, and 2) the inhibition of key enzymatic reactions by a myriad of substances which can severely impact the precision of absolute and relative quantitative gene expression analysis. PREXCEL-Q (P-Q) addresses these concerns head-on and automates and speeds up qPCR calculations (from hours to seconds) with precision, thereby eliminating human error and reducing reagent waste to a minimum. It also identifies and avoids inhibition of reverse transcription (RT) and qPCR reactions, identifies the valid LOG-linear-amplification-capable ranges for all target standard curves, calculates the valid dilution series for each nucleic acid sample on a per target basis, and is able to achieve nearly 100% reaction efficiency for most final qPCR target reactions (assuming appropriate primer-probe designs are already in place). There is no other similar comprehensive program. The current Excel 2003-based version of P-Q is increasingly being used by laboratories at Iowa State University, other American universities, in the United Kingdom and in other places across Europe. Our current efforts are focused on building a graphical user interface (GUI) for the program by converting the Excel 2003-based P-Q version entirely to Java for universal computer access, evaluating the developing GUI via target user feedback, documenting the GUI code, and creating a highly intuitive user manual and interactive Iowa State University-based P-Q website. A P-Q output file for liquid handling robots will also be developed. Our long-term goal is to make P-Q easy to use and easily accessible to all laboratories performing qPCR across the world (2).

## INTRODUCTION

The principles which underlie the successful execution of qPCR invariably center on initial sample preparation. But, in addition to this, and of no lesser importance, consistency of technique, from the very beginning to the very end of each assay is required so that all experimental samples are given the best possible, most scientifically-plausible chance to truthfully reveal the secrets they conceal.

Common difficulties that have persisted over the years with qPCR include: a) the time involved in correctly performing nuclease treatments, reverse transcription reactions, kinetically-correct sample dilutions and master mix assemblies for the assays, and b) inhibition of the [RT and/or Taq] reactions by a myriad of substances. These problems have caused many laboratories to take short cuts or to perform assays with a false sense of confidence (i.e. not knowing if or when RT and/or Taq polymerase inhibition is present). There is often also a tendency to pull back on assay complexity because of the tentative experiences investigators first have with qPCR. P-Q is an operational software program that can perform all concerned qPCR-related calculations in seconds or minutes. In our hands, it has removed the danger of performing meaningless assays for days, weeks or months. In addition, it assists the user in attaining optimal sample, target and standard dilutions which avoid sample-related reaction inhibition and allows qPCR reactions to consistently attain high amplification reaction efficiencies. P-Q also establishes/suggests the [standard practice] use of a “Stock I” solution for all qPCR studies - which will serve to ensure the uniformity of all qPCR approaches and assessments, and therefore greatly improve the ability of different labs to consistently cross-corroborate important gene expression results from micro-array and additional qPCR studies. Further, the use of “Stock I” to identify and avoid working within the inhibitory range of the assay provides a means by which investigators can arrive at the same results as those generated by correlate Northern Blot analyses, albeit with much greater sensitivity per unit sample amount.

### Background

**Basics of qPCR.** Real-time polymerase chain reaction, also termed “quantitative real-time PCR” (qPCR) is a technique used to amplify and quantify a specific portion of a DNA (or cDNA) molecule. The DNA of cells is composed of sequences that code for specific proteins. For this to occur, DNA is transcribed to form messenger RNA (mRNA) which, after appropriate nucleolar- and spliceosome-related processing, is transported to the cytoplasm where it is translated into functional protein at the ribosome. The number of copies of mRNA transcribed from DNA roughly correlates with the amount of functional protein formed. Therefore, being able to quantify the number of copies of mRNA (mRNA level) provides information on: 1) the extent of transcription of a specific portion of DNA, and 2) the potential amount of functional protein. Traditionally, mRNA levels were determined by Northern Blot analysis; however, this technique requires relatively large amounts of RNA and cannot be performed on limited, small or partially degraded RNA samples. By contrast, with qPCR, a small specific stretch of RNA is converted to complementary DNA (cDNA) by an enzyme called reverse transcriptase (RT), and specific regions on target cDNAs can then be amplified by *Thermus aquaticus* (Taq) DNA-dependent DNA polymerase by a process known as the polymerase chain reaction (PCR) (22, 23). Using fluorophores, each round of DNA amplification can be detected and measured as it occurs, in “real time.” With TaqMan hydrolysis probe-based qPCR, amplification of specific regions on the cDNA is guided by primers (which are smaller stretches of synthesized DNA that bind known specific stretches of the target DNA or cDNA sequence; each with a T_m_ of ~60°C, typically). Between these sites, a synthesized fluorescent-capable (TaqMan™ hydrolysis) probe, specific to a portion of the intervening target sequence, also hybridizes (such probes are designed with T_m_ values typically ~10°C higher than the primers so that they bind to target sequence first, before the primer(s) do, ensuring that all specific amplification events will be reported). The target DNA is amplified between (and including) the primers by Taq polymerase which (by its unique 5’-3’ exonuclease activity) sequentially degrades (hydrolyzes) the probe from the 5’ end, displacing the nucleotide containing the fluor from the formerly intact probe. Once this fluorescent portion of the probe is released and is no longer proximal to the quenching molecule (or quenching processes which are not dependent on exonuclease cleavage (10); see also the excellent animations by Biosearch Technologies at http://biosearchtech.com/download/flash_guides/formats_explained.html) designed into the intact probe, it fluoresces, and the photomultiplier tube of any commercial qPCR machine can detect its characteristic fluorescent signal wavelength. Geometric increase of the fluorescent signal corresponds to exponential increase in amplified sequences that, on a logarithmic scale, is linear and directly proportional to the initial amount of target sequence.

The cycle at which the signal rises above “background” threshold is termed Ct (for “threshold cycle”) or CP (for “crossing point”). Most qPCR machines require about 10^10^ (ten billion) copies before the accrued fluorescent signal is able to cross the threshold of detection (~10 standard deviations above background). Thus, at an amplification efficiency of 100%, the earliest qPCR signal from 1 copy of target nucleic acid should theoretically cross threshold at approximately cycle 33.22. But, due to the random ability of primers, probes and Taq to find single copy targets and initiate PCR in the reaction tube or well, an approximate Ct spread of 34.37 ± 1.15 would be expected. Stochastically speaking, 1 copy should always be able to amplify (in the absence of inhibitory phenomena), provided that the tested sample indeed contains the single target copy in the first place. The cycle at which the amplification first starts for such a single copy sample, however, has been experimentally observed to vary 5.5 cycles or more (34); and such sporadic Ct values are emblematic of what is called the “Monte Carlo effect” - but this is expected in terms of the *Poisson probability distribution* of such events; e.g. Lockey *et al*. observed that, for 30 samples thought to contain at least one copy of target transcript, 63.2% of the samples would be expected to actually contain one or more copies while 36.8% of them would be expected to contain no target at all (10, 34). In other words, the random nature of PCR amplification reactions in general is exacerbated the fewer target molecules there are in an experimental sample to begin with going into the reaction (10). But, in samples containing 10 or more starting copies of target, the ability of qPCR to generate reliable signals improves rapidly and reliably (given an absence of RT and qPCR inhibitory phenomena) (10, 34). Since the quantity of each specifically-amplified DNA (amplicon) sequence doubles every cycle (at 100% efficiency), and since the rate of amplicon accumulation is directly proportional to the initial starting amount of each specific target sequence in each sample, each target can be quantified and compared to a standard curve containing dilutions of known or relative amounts of target sequence, and the measured amount of signal from a gene of interest is finally divided by the signal measured from a reference gene or the geometric mean of several reference genes (which are assumed to exhibit steady-state gene expression within cells) in order to correct for “sample loading” from sample to sample (an assumption that has become less and less acceptable over time and which underscores the importance of responsibly choosing appropriate, valid reference genes for the specific sample set at hand). Further, becoming more aware of the math that underlies qPCR can be very helpful when interpreting Ct values generated from unknowns and/or serial dilutions of a sample or sample mixture. For example: a) d_n_ = {[1/LOG_2_(E_AMP_)] - 1} tells the investigator how many Ct units per cycle each successive dilution in a progressive dilution series will be expected to be off from the ideal expected occurrence of Ct values (e.g. when efficiency of amplification (E) = 100%; or E_AMP_ = 2); b) LOG_EAMP_(serial dilution factor) = expected Ct frequency between successive samples in that serial dilution progression; c) Ct_observed_ ± LOG_EAMP_(serial dilution factor) = the next expected Ct in that dilution series; d) 2^(ΔCtideal/ΔCtobserved)^ = E_AMP_; e.) Relative quantity for any target = 10^[(Ct-b)/m]^, and so on. An intercept-independent approximate equation for calculating the initial number of copies of a target (X_o_) from any Ct when m is known can be expressed as: X_o_ = 10^((Ct/m) + LOG_10_(Nt))^ or X_o_ = 10^((Ct/m) + 10)^, wherein N_t_ is the number of target amplicons generated at 100% E (E_AMP_ = 2) at the threshold of detection (Ct) from the perfect amplification of 1 initial copy. This Ct value should theoretically occur at 33.219 (or 10*[LOG_10_(2)^-1^]) cycles: 1 copy*2^33.219 cycles^ = 10^10^ copies = N_t_ = X_n_ = X_Ct_ = number of target copies at Ct. Thus, 10^10^ theoretically remains constant at any Ct for any target evaluated at the same fluorescence (ΔR_n_) threshold fixed at approximately 10 standard deviations above assay background. Interestingly, an additional, more universal equation emerges for calculating X_o_ in all cases where sample and standard material are experimentally “identical” (in keeping with the *Stock I concept* that this work encourages). This equation relies only three values: 1) a target’s Ct value; 2) the E_AMP_ value for that target (as estimated from a Stock I-derived standard curve); 3) the threshold value (T) at which the Ct value was obtained (using a ΔR_n_ scale ranging from 0 to 1; T values on this scale typically fall between 0.01 and 0.5 in practice). Briefly:

AX0=TΔRn1011∗∗EAMP−Ct

This expression, named the *Gallup-Overstreet equation*, revealed itself in conjunction with a series of intensive qPCR experiments performed by Anne-Marie C. Overstreet on eight ASF bacterial strains in mice. When qPCR amplification curves are assessed using y-axis (reaction fluorescence) scales that yield T values > 1 (36), the above approximation can be re-stated:

BX0=FCt/Fmax1011∗∗EAMP−Ct

When the “10^10^ amplicons at a ΔR_n_ or F_Ct_/F_max_ threshold of 0.1” assumption does not apply, the following threshold-independent equations (which allow the calculation of a y-intercept in terms of copies for experimental sample-derived relative dilution standard curves from the y-intercept evaluation of a corresponding absolute target template standard curve) can be applied:

C Absolute template:Xo=10−ba/maEAMPa−Cta∗

D Sample template:Xo=10−bs/msEAMPs−Cts∗

Here, the term “b_*s*_” in equation (D), is the *calculated copy number-associated y-intercept* of the experimental sample-generated plot of: *LOG of target copy number vs. Ct* (not to be confused with the y-intercept obtained directly from the plot of: experimental sample-generated *LOG of relative sample dilutions vs. Ct)*. b_*s*_ is mathematically obtained as follows:

Ebs=baLOGEAMPa/LOGEAMPs∗

This transformation assigns an appropriate copy number estimate to the calculated y-intercept value for the copy number-transformed relative-dilution target standard curves. However, when absolute standards are not used, above approximations (A) or (B) can be applied - but only cautiously, as they assume a stable (10^10^) number of amplicons to be generated at a threshold of 0.1 (37, 38).

Currently, qPCR is commonly used in life science laboratories in work ranging from microbes to plants as well as in medicine including human and veterinary. It is also a workhorse assay for diagnostic and forensic samples. However, although qPCR is informative on many fronts, leaders in the qPCR field have responsibly warned investigators and researchers to temper their enthusiasm over qPCR results by remembering that “*qPCR data constitute only a snapshot of information regarding the quantity of a given transcript in a cell or tissue. Any assessment of the biological consequences of variable mRNA levels must include additional information regarding regulatory RNAs* [miRNA, shRNA, siRNA], *protein levels and protein activity*” (29). In addition, the possibility that there are splice variants of every message investigated must always be taken into account (10, http://lane.stanford.edu/howto/index.html?id=_2063 and http://medblog.stanford.edu/lane-faq/archives/research_publishing/index.html).

**Figure 1:**
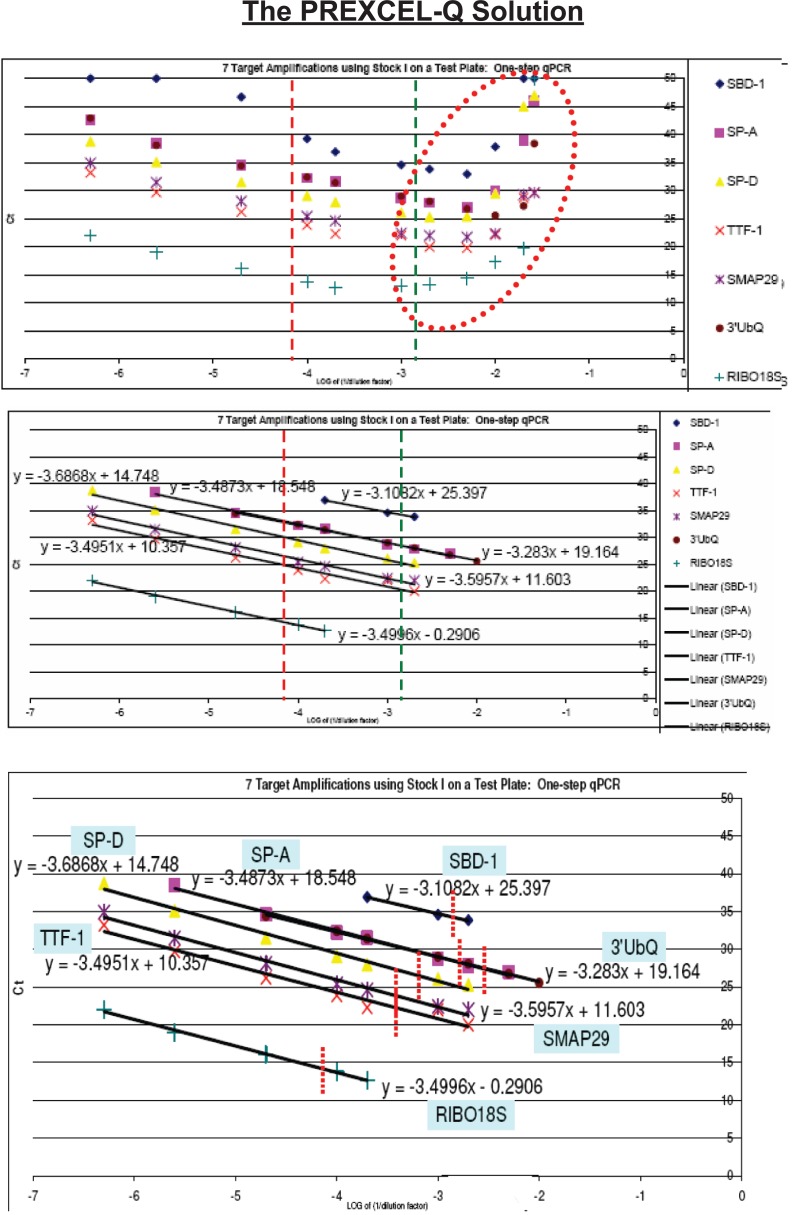
qPCR-inhibitory behavior (top graph) of a Stock I solution at different dilutions. The qPCR data shown was collected for 7 targets of interest in ovine lung tissue from a P-Q Test Plate analysis preceding final qPCR set-ups for a 56-sample experiment. Ct results generated at LOG-linear-amplification-capable sample dilutions exhibit a straight line (middle graph), while the inhibitory dilution range is curved like a hook at lower sample dilutions (top graph). The red dotted oval encircles this “hook” portion within which most of the dilutions are inhibitory for each of the qPCR amplifications of the transcripts of interest. Within these inhibitory sample dilution regions, investigators will obtain results (Ct values); however, they will be wildly misleading and incorrect. For example, a sample diluted within the inhibitory dilution range will generate a target Ct that can be directly mistaken for a non-inhibited sample that actually has a low amount of that target (see Figure. 2). To the left of the red circle, where samples are more dilute, Cts become LOG-linear. Note that targets can differ for each of their optimal LOG-linear-amplification-capable dilution ranges (bottom graph). Therefore, it is not accurate to simply dilute all samples to 1:200, for example. With some target transcripts, we have found optimal dilution ranges from 1:250-1:5000 (e.g., SBD-1) to 1:4000-1:4,000,000 (e.g., RIBO 18S) within the same Stock I. P-Q identifies these precise LOG-linear-amplification-capable dilution ranges for each different sample and target, and the entire process for calculating these parameters for 7 targets is rapid (15-30 minutes) with P-Q.

**Figure 2:**
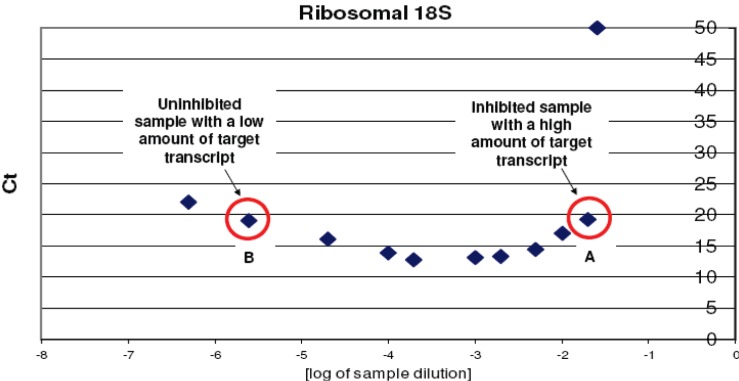
The danger of not working outside the sample-inhibitory range of qPCR assays. This is a Stock I sample mixture that has been diluted in order to identify the LOG-linear-amplification-capable range for 18S ribosomal RNA (18S rRNA). Note that dilutions of Stock I with high and low amounts of 18S rRNA transcript can be mistakenly interpreted (if they were unknowns) as containing the same amount of 18S rRNA target message since they both generate virtually identical Ct values depending on the degree of inhibition present or absent at different sample dilutions. Both low dilution A (high amount of target transcript) and high dilution B (low amount of target transcript) generate the same Ct value (~19). P-Q avoids this problem with every sample and every target for each sample.

Technically, there are several basic considerations for setting-up qPCR assays which have been discussed at length in the literature (3). These include: a) decisions by the operator regarding design and concentrations of primers/probes, choice of the appropriate master mix (some containing both reverse transcriptase and Taq DNA polymerase, and others, just Taq DNA polymerase) to carry out either One- or Two-step qPCR; b) RNA isolation and cDNA synthesis; c) selection of appropriate/stable endogenous reference gene(s) or exogenous control sequence(s); d) determination of reaction efficiencies and valid dynamic dilution ranges for each target standard curve; e) kinetically-appropriate sample dilutions; f) correctly handling and processing samples derived by laser-capture microdissection (LCM); g) appropriately designing Test Plates. P-Q addresses each of the above (with the exception of primer design), allowing investigators to step almost immediately into the lab to commence with qPCR since all set-up calculations can be optimally performed by the program in minutes – as opposed to hours, days, weeks, months, even years otherwise spent in vain (Figs. 1 and 2).

### Key problems with qPCR: Set-up calculations and inhibition

**Calculations.** As indicated, there are assay-specific key calculations required to perform qPCR correctly, some of which (e.g. appropriate sample/target and standard dilutions) are often entirely ignored (2, 4, 5, 6, 7, 8, 9). After completing the calculations, laboratory personnel then need to perform pipetting procedures that can introduce additional, compounding errors when the pipetted amounts of each reagent and/or sample are not planned out (and printed out) clearly beforehand. These are some of the commonly-understood real-life concerns with qPCR. Not only has P-Q reduced our perfunctory calculation time from hours to seconds or minutes, its recipe-like output reports greatly minimize the occurrence of user-introduced procedural set-up errors. Most importantly, P-Q identifies and addresses the often ignored problem of sample-related inhibition that is likely commonplace in every laboratory performing qPCR.

**Inhibition.** A variety of problematic inhibitory phenomena has been reported that plague qPCR assays (2, 4, 5, 6, 7, 8, 9). Inhibition of the enzymatic reactions involved in generating real-time qPCR signals from specific cDNA templates using specific primers, fluorogenic probes, or combinations of primers and fluorogenic probes can severely influence the precision of absolute and relative gene expression quantitative analysis. Any factor, experimental, user introduced, environmental or otherwise, that has an impact on the activity of RT (reverse transcriptase) enzyme and/or Taq DNA polymerase used in any real-time qPCR reaction will invariably affect the results generated. In worst-case scenarios, these deficiencies go unnoticed, remain unaddressed and end up published as experimental “results.” Recently, others have suggested that many as yet unidentified sample-specific substances (or impurities) are often carried over as a result of different RNA isolation methods (preceding real-time qPCR of any variety) which cause RT enzyme- or Taq DNA polymerase-based qPCR inhibition (9, 10). Exogenous contaminants such as glove powder and phenolic compounds from the extraction process and plastic-ware (pipette tips, tubes and plates) can also have an inhibitory effect. With regard to tissue-specific inhibition of DNA amplification, *tissue type* was found to be the largest source of variance of inhibitory phenomena while primer sequences appeared to have the least affect. In other words, tissue type from which total RNA was extracted had the most significant effect on PCR kinetics, thus on final threshold cycle (Ct) values (9, 11). This is thought to be caused by different kinds and amounts of cellular debris present in samples after RNA extraction (9, 10). Endogenous contaminants such as blood or fat are thought to play an important role in affecting both the PCR as well as the preceding reverse transcription reaction. Other inhibitory contaminants are thought to be hemoglobin, heme, porphyrin, heparin (from peritoneal mast cells), glycogen, polysaccharides and proteins, cell constituents, Ca^2+^, DNA or RNA concentration, and DNA (and possibly RNA) binding proteins (12-18). MicroRNA (miRNA) is not thought to be a contributing factor to qPCR inhibition since high thermocycling temperatures (94-95°C) most likely prevent the formation of stable RNA-binding (RISC) complexes which might otherwise associate with template RNA. Inhibition is especially a problem with ancient DNA retrieved from archeological samples or other partially-preserved organisms that have either degraded or amassed qPCR-inhibitory contaminants over thousands and tens of thousands of years (4).

### Test Plate assessment of a representative mixture of samples: The “Stock I” solution

A common problem with qPCR is selecting a sample that is representative of all samples in a study (to control for sample source variation, method of sample isolation, DNase treatment, RT conditions and serial dilution effects, etc), and a source of material to use for qPCR target standard curves. When completing a study with tissues from several mice or other animal species or even cultured cells, where does one obtain an appropriately representative RNA or DNA sample to test all of the genomic or transcriptomic targets of interest in each qPCR study? Some investigators pick one representative sample (e.g. Roche Applied Sciences “E-Method” (33)) while others use transcribed or reverse-transcribed linearized plasmid constructs as the representative test sample and as the standard curve material. We, however, pool small amounts of all or some of each current group of experimental samples and use the resulting mixture as the overall representative sample in each of our qPCR studies; we have named such mixtures of experimental samples, “Stock I.” Stock I serves both as the serially diluted sample for each qPCR study’s preliminary “Test Plate” (which allows for the most realistic assessment of the qPCR dynamics of each sample and target of interest - the results of which are subsequently used/interpreted by P-Q to precisely define the valid dynamic boundaries of each entire qPCR study, per sample, per target, per isolation-method) and as the standard curve material on all final qPCR plates for individual sample/qPCR target assessments. This is an original (yet obvious) idea which, we feel, offers an excellent approach to qPCR which will help it achieve a consistent level of precision from lab to lab. Since the idea and use of a “Stock I” solution (which we introduced in 2001 and first published in 2004) (19) directly combats the only valid criticism of the Ct method by Sigmoidal Curve Fitting (SCF)-method proponents (e.g. standards and samples often not being comprised of the same material), (20), we have offered a viable solution to a dilemma facing qPCR users world-wide (the vast majority of whom will likely use Ct-based qPCR analysis for years to come). P-Q can also be used without the ‘Stock I solution’ option. Others have also recently observed that the “SavrgE^Ct^” method (which P-Q essentially accomplishes by virtue of its use of “Stock I”) is actually much more precise than any SCF-based method for qPCR quantification. P-Q therefore theoretically attains the same precision, accuracy and robustness as the “2^-ΔΔCt^ Method” when target amplification efficiencies are ~100% (21, 35).

### The PREXCEL-Q Program Description

The P-Q program provides a simple, universal and standardized approach to qPCR which was first described by the authors in 2006 (2) (see also Appendices 1 and 2). Out of respect for those who have invented this brave technique (Mullis, Higuchi; 22, 23), this program is offered as a responsible follow-up implement which endeavors to ensure the fidelity of qPCR execution across the world. The future of P-Q involves its conversion to a Java-based graphical user interface (GUI)-driven program. It currently resides and works best in Microsoft Excel version 2003. Windows Office 2007 Excel and Macintosh platforms are not generally able to run the program, although Macintosh computers loaded with Windows Excel 2003 (e.g. using Parallels 3.0) can be used to run the program in a Macintosh environment. P-Q’s restriction to Windows Excel 2003 is a little disconcerting, but Excel 2003 is still largely available at most universities and government agencies using qPCR throughout the world. But, indeed because of this, the program’s conversion to a universally-accessible Java-based GUI format remains top priority for us as computers and their associated operating systems will most certainly continue to evolve. Empirically, P-Q is a collection of 27 interlinked Excel files commandeered by interwoven visual basic (VBA) macros and extensively layered, interdependent algorithms. The user interacts with mainly 5 of the 27 program files. Additional helpful Excel files (for sample preparation, DNase treatments and quick master mix set-ups) are also provided with the program - files that can be used in cases where the program in its entirety is not called for. P-Q is Ct based. It relies on the threshold cycle (Ct) values generated on a Test Plate for the remainder of its functionality. Its goal is to help generate trustworthy Ct values, and, once these values are generated reliably, investigators can then process them with confidence.

### Test Plate

The initial goal of the program is to establish a *Test Plate* (or validation plate) set-up using a representative sample mixture (made of a small portion of some or all of the experimental samples in each qPCR assay) to assess up to 7 targets of interest (per each instance of the program) over a carefully selected progressive dilution series of the chosen sample mixture. This sample mixture, we have arbitrarily named “Stock I” If investigators choose not to use the “Stock I approach,” the P-Q program can be used that way as well.

### Required User Input

First, from the user, the P-Q program needs the input of 7 basic parameters either into the program’s “Questionnaire.xls” file, into the “UMES.xls” file, or into both files: 1) Sample-preparative information entered into cells I134 and F135 of the “UMES.xls” file and into “Questionnaire.xls” file cell range C6:M37, 260_nm_ readings of all RNA or DNA samples at a known dilution (the factor for which is entered into “UMES.xls” file cell P137 and into “Questionnaire.xls” file cell G30 - which receive a value of “1” for samples measured at no dilution/full-strength; e.g. as with many NanoDrop readings) entered into “UMES.xls” file cell range B120:B191 along with the singular selection of the appropriate template extinction coefficient in cell range R150:R153, and selecting (using an “x” in cell range D120:D191) which samples will contribute to “Stock I,” and how many μL each sample will contribute to the creation of Stock I (which is either entered manually or entered automatically as calculated by the program) into “UMES.xls” file cell F2, in addition, enter sample size prepared for the Test Plate into cell “UMES.xls” cell K2; 2) Exact knowledge (parameter entries) of the conditions used for DNase or RNase treatments (these should be ‘identical’ for each sample) entered into cell range F138:F139 and K136:K139 of the “UMES.xls” file and within cell range G42:H57 of the “Questionnaire.xls” file; 3) Exact knowledge (parameter entries) of the RT (reverse transcription) reaction assembly (these should also be ‘identical’ for each sample) entered into cell range N120:N121 and P134:P135 of the “UMES.xls” file and within cell range P53:H79 of the “Questionnaire.xls” file; 4) The stock concentration of the primers and/or probes (all diluted to the same concentration; generally between 2 and 10 μM for singleplex qPCR as entered into “UMES.xls” file cell M117 and between 40 and 100 μM for multiplex qPCR as entered into “UMES.xls” cell S157) and the final in-well^a^ nanomolar (nM) concentrations the primers/probes are each to be used at entered into “UMES.xls” file cell range H126:J132, with species and target names entered into cells F126:G132 adjacent to that same region; 5) Any pre-knowledge pertaining to the relative abundance of any or all of your targets of interest (e.g. if one already knows that the targets of interest are “loud/robust” (relatively high-copy) signals in your samples and in your qPCR assays, testing Stock I dilution ranges from full-strength out to 1:1,000,000 dilution (in-well) or greater for the Test Plate is justified. If the targets are known and/or thought to be relatively rarer, one should test a less extensive Stock I dilution series (e.g. from full-strength out to 1:100,000, 1:50,000 or 1:10,000 or less) to get an accurate preliminary picture of each sample’s qPCR targets’ behavior over an appropriately informative dilution series. Generally, a serial progressive dilution series ranging from full-strength out to 1:100,000 works in most situations (except for LCM nucleic acid isolates); 6) Drawings of all final plates, and a good preliminary manual estimate of how much of each sample and standard will be required, and how many technical replicates will be used for samples, standards, NTCs, NRC/NAC samples (values for which are entered into “UMES.xls” file cells F55, F58, J17, J19, J20, J24, O25, U25 and AA25, respectively) and, 7) User’s choice of either One-Step, Two-Step or LCM-qPCR and specific knowledge of the relative strengths of the existing components provided with the particular Master Mix product(s) to be used (e.g. 2X, 2.5X or 5X Master Mix [with or without ROX], 50X, 40X or 25X RT or RT-Taq solutions etc., the μM concentration of the ROX stock solution and the mM concentration of the MgSO_4_ or MgCl_2_ stock solutions provided in the particular kit being used). [Upon reset, the P-Q program defaults to using ABI’s One-Step Master Mix Reagents Kit which includes a 2X Master Mix and a 40X RNasin-RT solution].

The power of foresight allows one to choose the extent of dilution which is most aptly suited to the target set of interest during each Test Plate analysis. Using conditions tailored specifically to any pre-knowledge of the relative abundance of each of the target transcripts is a crucial stratagem to harness/employ during Test Plate designs. If the tested Stock I dilution range is not sufficiently wide, one may miss the opportunity to have assessed a wider [valid] dynamic range on the final experimental sample plates; this can be the consequence of not exploring more extensive dilution series’ on one’s Test Plates. Conversely, using too wide of a Stock I dilution range on a Test Plate can make it hard to identify the Stock I dilution(s) at which certain qPCR target signal strengths begin to die off. Also, it is important for investigators to know when it is prudent to use either median dilution ranges or differential Stock I dilutions (on a per target basis) when the targets of interest are thought to differ greatly in relative abundance (in Stock I) with respect to one another when being assessed on the same Test Plate. Sometimes running more than one Test Plate may be necessary to *get all* the preliminary information you need.

The P-Q program has been largely created to use pre-formulated, commercially-available Master Mixes (in effort to maintain the “high-throughput and reproducible” philosophy so prevalent in the qPCR, micro-array, mass-array and proteomics worlds today), but it can also be used in conjunction with self-made/custom Master Mix formulations if one is able to translate such custom mixes in [relative or direct] terms of one of the commercial qPCR mixes already included among those whose assembly is automatically spelled out by the program. The pertinent Master Mix parameters are entered into “UMES.xls” file cells G31 and G32 (and additionally into cells K197, L199 and N202 for other required Stratagene Master Mix parameters). Final prepared qPCR reaction parameters (e.g. sample volume used per each prepared qPCR reaction) are entered into cells N134, N136 and N138 of the “UMES.xls” file and into cell ranges H87:K98 or F109:K120 of the “Questionnaire.xls” file (singular qPCR reaction component entries are available there as well). Differential use of MgSO_4_ and/or MgCl_2_ on a per-target basis is also adjustable (in terms of Invitrogen Master Mix) - inside the “zPrintouts.xls” file (cells AR15, AW15, AR24, BA23, BA24, AR33 and AR42 of the “TP MM”, “SP MM” and “NRC MM” sub-worksheet ‘tabs’ within that file). In addition, ROX usage is also adjustable in various regions of the “zPrintouts.xls” file for some of the various Master Mixes shown. Transposing specific formulations of one Master Mix in terms of another Master Mix (those not included in the program) is up to the investigator as it is understandable that the program cannot include or account for all possible Master Mixes. And, in the event that one enters impossible primer or probe amounts, the maximal allowable amounts for primers and/or probes is shown within specific regions of the “zPrintouts.xls” file to guide users to precise and correct set-ups.

### PREXCEL-Q sample number limitations

Auxillary program file, “01AuxSmplFile.xls” allows an infinite number of RNA samples to be processed in a One-Step qPCR application. And, while only 60 samples are allowed at a time for Two-Step qPCR (where RT reaction formulations are to be shown by the program), if cDNAs have already been made, P-Q can again be used to handle an infinite number of samples. A maximum of 7 targets is allowed at a time. For more than 7 targets, use “Quick Mode #6” or merely use multiple instances of the program (renaming the master folder each time it is to be copied and the program used for another set of targets). The accompanying P-Q user’s manual can be consulted for more detail on key aspects of the program.

### GOAL #1: Create a Test (validation) Plate using “Stock I”

The program suggests the use of a “Stock I” mixture (a mixture of a portion of each of your experimental samples) to serve as the serially-diluted sample used on the Test Plate, and doubly, as the material from which all standard curves will be made on your final experimental plates. To justify this approach, several general assumptions have been made:

Theory: “Stock I” will behave most identically to your actual samples since it is made of the very samples themselves.

Theory: Purified plasmid constructs cannot be assumed to behave similarly to tissue, cell or other biologically-derived nucleic acid samples unless they are spiked into experimentally similar samples early on and subjected to the same regimen of nucleic acid isolation/purification as the experimental samples have been. In addition, many plasmid constructs are not currently available. Nonetheless, the genesis of all sample, standard and/or calibrator isolates should be kept as similar as possible; an approach that is accepted as a “good science” practice in general. All qPCR methods benefit from the use of similarly-prepared samples and the use of standard curves that are truly suited to and/or substantively representative of the samples being studied. Standards can, however, also be made from any appropriate amplifiable nucleic acid material but should be used only for samples which have all been prepared identically (10, and a March 01, 2006 Drug Discovery and Development Webcast @ http://www.dddmag.com/reliability-of-qPCR-data.aspx).

Theory: To be safe, it should always be assumed that all samples contain unintended carryover material(s), molecules or chemicals, some of which are inhibitory to the RT phase, Taq (PCR) phase, or both phases of the qPCR. Every different nucleic acid isolation method and sample type from which nucleic acids are isolated has the potential to introduce inhibition of some kind. Also, it should never be assumed that column purification methods used for nucleic acid sample isolations are more effective at eliminating inhibitory materials. Some of the *worst* qPCR inhibition we have observed occurred among samples isolated using column methods (1:3000 CJ, 1:2000 MC; Dr. C. Johnson and M. Carruthers, unpublished results), but, as well, some of the *least* inhibition we have seen occurred among samples isolated/purified using column methods (1:50 SC, 1:150 ZP, 1:30 NAL, 1:60 EB; Dr. B. Sponseller and S.K. Clark, Dr. Z. Liu, Dr. N.A. Levy and Dr. E. Behlke, unpublished results).

**For Absolute qPCR,** the ng/μL values generated by P-Q can be readily converted to copy numbers by users whenever the relationship between absolute standard material and copy number per unit volume or unit template mass is known. Nevertheless, evaluation of Stock I material (made of the samples) is still necessary in order to provide the user with the exponential amplification (E_AMP_) values for each target when amplified from sample material since E_AMP_ values for these same targets when amplified using absolute template material (e.g. purified plasmid(s) containing target insert(s)) will differ (usually be higher) due to either purified absolute template material harboring less inhibitory material, or due to plasmids (in general) often amplifying by PCR with higher efficacy than other target template varieties on account of geometry alone. So, when one knows the concentration of absolute template material in terms of both ng/μL and copies/μL, and after one has compared the absolute template’s ability to amplify for a target with that of a sample or Stock I’s ability to amplify for that same target (wherein all such measurements have been made within the valid, non-inhibitory, LOG-linear-amplification-capable, high-efficiency-of-amplification Stock I and absolute template dilution regions), the magnitude of difference between these two evaluations of the same target yield an attenuating factor which is entered directly into P-Q for each different target standard curve graphic as shown in the program’s “zPrintouts.xls” file’s “Qspecs” worksheet tab. Attenuating factors (for each different target) are entered into “Qspecs” cells: AH22, AU22, AH63, AU63, AH103, AU103 and AH147. Once you have calculated how many copies of target there are per μL qPCR reaction for both Stock I and absolute template material, the calculated number of target copies/μL contained at each Tier ng/μL dilution of Stock I material are entered into “Qspecs” cells: AI27, AV27, AI68, AV68, AI108, AV108 and AI152 for each different target. “Qspecs” cell range AX40:BJ79 provides the entire network for up to 7 targets for exacting these calculations. Only one valid target Ct observation per absolute template material in terms of copy number/μL qPCR reaction paired with one similarly valid observation for the same target using Stock I material is necessary to estimate copy numbers of that target in unknowns/samples. As a result of these “Qspecs” entries, the program shows the investigator the equivalent ng/μL for each standard to which the absolute template material for each target should be diluted to kinetically encompass the same valid, LOG-linear-amplification-capable dilution range exemplified by each target when amplified from within sample or Stock I material. The equation we developed to calculate each target’s attenuation factor is as follows:

Equation 1: ((Sample-derived template target’s E_AMP_^observedCt^) / (Absolute template target’s E_AMP_^observedCt^))*(Sample derived template ng/μL evaluated in-well/Absolute template ng/μL evaluated in-well) = Attenuation factor (the factor by which absolute template is diluted to exactly/kinetically mimic sample-derived Stock I for a specific target)

Finally, it logically follows that Stock I-attenuated absolute template (plasmid) mixtures would use the same 260_nm_ value as the Stock I mixture itself (e.g. entered as a ‘surrogate/stand-in value’ into “UMES.xls” file cell J1). This is a rarely used, but helpful P-Q feature.

**Use of exogenous Stock I mixtures.** Made of (newer or older) samples other than the ones being evaluated presently, is also possible. In such cases, the investigator will enter the actual or calculated 260_nm_ absorbance reading of the exogenous Stock I material directly into “UMES.xls” file cell J1 and proceed to use the program as usual. Along with the desired prerequisite that externally-introduced Stock I solutions be comprised of material that has experienced the same genesis as the experimental samples have (and for which it will be used as a measure), it also becomes clear that it is intuitively best to dilute such Stock I solutions to the average ng/μL of what a Stock I solution would be if the samples themselves had been mixed equivolumetrically - in “normal Stock I fashion.” Assuming that the “Stock I” idea will be pursued (one which we are avid proponents of for numerous, first-hand experiential reasons):

After all required entries have been made (within the “Questionnaire.xls” and “UMES.xls” files), the next step toward arriving at the Test Plate set-up is to decide what dilution range of the “Stock I” material is best suited to the particular genes or transcripts of interest. This is accomplished by using what is known about the expected relative abundance of the targets of interest in conjunction with the Ctrl Shift A Macro of P-Q to calculate the most suitable dilution series of “Stock I” to be tested across each of the different targets. Up to 7 targets per Test Plate are allowed. Typically, dilution ranges of full-strength to 1:1,000 are appropriate for most LCM-derived samples (of 25-500 cells per original sample isolate), full-strength to 1:10,000 dilution for non-LCM-derived rare targets or transcripts, full-strength to 1:50,000 or 1:100,000 for medium-abundant transcripts, full-strength to 1:1,000,000 for “normally-expressed” targets or transcripts, and full-strength to 1:5,000,000 for abundant targets. (For those of you who use RIBO 18S or RIBO 16S as a reference gene or organismal identifier, you will notice that, even at an in-well dilution of 1:5,000,000 or even greater, these signals are still reliably measurable. Imagine diluting 1 μL of sample into 5, 10 or 20 Liters of water and still getting robust Ct values! This speaks well for the incredible sensitivity of qPCR in general).

But, to continue, and to repeat a few important things, after one has successfully completed all of the required maneuvers described up to this point, and has 1.) Entered the appropriate Master Mix parameters into cells G31 and G32 (and additionally into cells K197, L199 and N202 for other required Stratagene Master Mix parameters if using a Stratagene Master Mix) or has run an auto-Master Mix parameter insert Macro (e.g. the two Master Mix parameter auto-insert Macros are Ctrl Shift D for Invitrogen Master Mix and Ctrl Shift O for BIO-RAD Master Mix), 2.) Entered the required target names and primer-probe [nM]-use information along with the target (‘t’) or reference gene (‘h’) designation for each within “UMES.xls” cell range J126:K132, 3.) Decided on the Test Plate Stock I dilution range, and has entered the desired appropriate upper Stock I dilution limit value in “UMES.xls” file cell M28, and 4.) Has activated Macro Ctrl Shift A, this is all immediately followed by running one of the three main user parameter introduction Macros:

- Ctrl F for One-Step qPCR (either “Questionnaire.xls” file-based or not)
- Ctrl x for Two-Step qPCR (either “Questionnaire.xls” file-based or not) then immediately double-check “UMES.xls” cell region O134:U138 for helpful messages.
- Ctrl w for LCM-based qPCR (use default One-Step settings, or use a “Questionnaire.xls” file-based or non-“Questionnaire.xls” file-based Two-Step approach if cDNAs are to be made and 260_nm_ values of the LCM RNA samples are known - or good estimates thereof at least). Some good approximate figures to keep in mind (for mammalian cells): one cell contains ~6.16 pg DNA, ~20 pg total RNA, and ~0.5 pg mRNA.

Next, if pursuing Two-Step qPCR, the investigator first sets up the sample RT reactions as acknowledged (and spelled out) by the appropriate printouts within the “zPrintouts.xls” file, and then sets up and runs the Test Plate according to the 3 printouts describing the Test Plate set-up in the “zPrintouts.xls” file, or, if the investigator is pursuing One-Step qPCR or One-Step LCM-qPCR (where RT reactions are not undertaken in a preliminary, separate step), the user sets up and runs the Test Plate according to the 3 printouts describing the Test Plate set-up in the “zPrintouts.xls” file.

### GOAL #2: Use the Test Plate Ct values to determine the non-inhibitory, LOG-linear-amplification-capable, highest-efficiency-of-amplification ranges for each target

This is accomplished using the “Point Selection Process” within files “TestPlateResultsAnalysis2006.xls” and “TestPlateResultsAnalysis2006b.xls” (after introducing the Test Plate Ct results into “UMES.xls” cell range B197:H208 and running the Macro Ctrl Shift C). Once the Point Selection Process is completed, and after introducing your final choices/selections/settings into the program using the universal system updating Macro, Ctrl Shift Z, one then checks for any error messages (in cells F3, E129 or BE9 of the “UMES.xls” file) and proceeds to correct them by either altering/correcting user input values, adjusting standard curve ranges, adjusting the “Sample Aiming Device” (in “UMES.xls” file cell range AX12:BK38) - all in conjunction with the strategic use of system-corrective/update Macros: Ctrl Shift Z mainly, but also Ctrl y, and Ctrl Shift N for certain Two-Step changes, and Ctrl Shift T for LCM-qPCR related changes, etc. All Two-Step methods default to no more concentrated than 1:50 in-well dilutions as the starting point for all standard curves for initial set-ups, so care should be taken to remember this and make your final desired adjustments after entering and processing Test Plate Ct values for Two-Step qPCR set-ups. The “Comprehensive Error Messaging Board” within “UMES.xls” file cell range J204:S227 lets the investigator know the source of all system errors (if any), and how to correct them.

### GOAL #3: Address any “Error” messages within the program

Once “OK” is received in the Error Messaging regions (in cells F3, E129 or BE9 of the “UMES.xls” file), you may want to prepare extra “Stock I” to use for additional studies (which can be accomplished by adjusting the value in cell F2 of the “UMES.xls” file and running Ctrl Shift Z, then check for error messages again, and re-check your plate drawings and your manual sample and standard volume requirement calculations to be sure your entries into cells F55 and F58 of the “UMES.xls” file are indeed appropriate (entering a little extra is always advisable). Update any changes with the appropriate Macro (again: Ctrl Shift Z mainly, but also Ctrl y, Ctrl Shift N for certain Two-Step qPCR-related changes, and Ctrl Shift T for LCM-qPCR related changes).

### GOAL #4: Attain your final sample plate and NRC/NAC plate set-ups

Run Ctrl y and Ctrl Shift Z one last time for good measure, and then proceed to identify and printout the appropriate pages within the “zPrintouts.xls” file and set up your final plates and NRC/NAC plates accordingly.

**Commercially-available qPCR Master Mix set-ups spelled out directly by PREXCEL-Q.** ABI Two-Step Master Mixes (Two-Step TaqMan and SYBR-based Mixes), ABI One-Step Master Mix Reagents kit, Invitrogen SuperMix with UDG, Invitrogen One-Step SuperMix, Qiagen One-Step Master Mix, BIO-RAD One-Step iScript Mix, Stratagene Brilliant One-Step Master Mix, and Stratagene’s Full-Velocity One-Step Master Mix. Any other Master Mix set-ups can be inferred from or are implicit within the formulations of one or all of those already spelled out by the program; e.g. be able to interpret/translate what the given P-Q set-ups mean in terms of the Master Mix you are using if it is different from the ones automatically spelled out by the program. Correlate SYBR-based mixes are implicit in each different company’s P-Q Master Mix printout. One can also use the Master Mix set-up numbers generated by P-Q as a guide to use self-formulated Master Mixes as well. Pre-prepared commercial kits exist which do not directly fit the output format of P-Q, but, their use, with some inductive input from the investigator, can be deduced via mathematical analogy from the various Master Mix set-ups already solved by the program.

**Appropriate mind-set when using PREXCEL-Q.** You will notice that the proper use of this program requires that the user works in a “projective” manner in which the final plate set-ups are envisioned at the same time the Test Plate parameters are being established (the beginning and end of the entire assay is in the mind of the user from the very outset; drawings of all desired plates, and manual approximate calculations of entire sample/standard needs are important for the investigator to already have in hand before using the program). The entire set-up from the Test Plate to the final plates and NRC/NAC plates are all calculated at the same time to check early on if there will be a problem with running short on sample, standard or Stock I material throughout the entire run. It also informs the investigator how much Master Mix, primers and probes will be needed to complete the entire procedure at hand and thus allows one to preview reagent needs before each particular entire set-up in order to avoid ‘false starts’ and to afford one the opportunity to place any orders for anything that may be in present short supply according to what is pointed out by the program in advance. The program tells the user whether or not there is enough of each sample, standard or Stock I material to finish the entire study in one cohesive fell-swoop. Doing the entire assay in one shot is also the only approach that can most fully guarantee scientific consistency throughout each entire qPCR endeavor. After Test Plate analysis and Test Plate Ct introduction to the program followed by the Point Selection Process, the program figures out the entire Master Mix and primer/probe needs for all final plates and NRC/NAC plates - and this can sometimes add up to 14 or 15 plates worth of Master Mix to be prepared all at once - but there is no better way to maintain data consistency than by using a common Master Mix pool for all reactions whose final Ct values are to be weighed with and amongst one another in effort to gain truly informative Ct values for generating meaningful relative or absolute qPCR data. Liquid handling robots are a blessing - get one or two if you can. Or, limit yourself to setting up only 2 or 3 plates at a time. We often do 14 plates at once - which typically takes one operator about 24 to 28 hours straight (manually, without liquid-handling robotics). Taking it in smaller pieces is better - but the Master Mix used on all such final sample plates should be the exact same pool.

### GOAL #5: Process your qPCR results using your own preferred approach

Creating custom Excel files based on the Pfaffl (or “E_AMP_^ΔΔCt^”) Method seems to work the best for targets exhibiting near ideal amplification efficiencies (e.g. 100% ± 15%). Also, remember that technical replicate Ct values should not deviate more than ~0.5 Ct units from one another in order that statistical analyses of qPCR data retain ample rigor.

## DISCUSSION

The P-Q approach to qPCR assumes, stresses, and requires consistency in all facets of nucleic acid sample preparation. This “good science” practice maximizes experimental sample similarity and, therefore, sample inter-comparability. Such facets are: a) Method of RNA or DNA isolation, purification and storage; b) DNase or RNase treatments and reverse transcription (RT) reaction formulations; c) Dilutions of all samples beyond the point where inhibition of RT and/or PCR is expected; d) Use of all samples, per qPCR target (outside RT- and PCR-inhibitory ranges) at the same ng/μL concentration within the valid, high efficiency, LOG-linear-amplification-capable dilution range per each sample (per target within each sample); e) Good primer and probe designs (hopefully designed using programs containing algorithms that are able to successfully identify and eliminate false priming regions from consideration).

P-Q re-introduces our (2004) idea of “Stock I” which can be a single sample, or a mixture of some or all of the samples involved in a qPCR study (19). This provides investigators a plentiful reservoir of material that is the best overall representative of how each individual sample will behave during RT and/or PCR, and which is used for preliminary target Test Plate analyses as well as the standard curve material for all final plates. Since all samples can contribute to Stock I, no single sample is exhausted prior to qPCR. NOTE: any standard curve can be generated ‘after-the-fact’ in cases where Stock I proves to be “anemic” or insufficient for a particular target or targets. Samples with ample signal for such targets are revealed individually on the final plates – after which custom Stock I sample mixtures can be formulated and standard curves run.

Always try to use maximal sample amounts in each Master Mix - why not? (Especially if you have plenty of each sample). This helps stave off the Monte Carlo effect as well. P-Q defaults to using sample maxima within each different company’s Master Mix. e.g. 7.2 μL/30 μL reaction for Invitrogen One-Step Master Mix or 7.8 μL/30 μL reaction for ABI and BIO-RAD One-Step Master Mixes (e.g. as initially inserted by the Ctrl Shift D or Ctrl Shift O Master Mix parameter auto-insert Macros, respectively; parameters which are still adjustable by the user afterwards).

On account of what P-Q does, there is theoretically no need to correct for “sample loading” (using reference genes) once you have proven you are working in the valid, LOG-linear-amplification-capable dilution range for each of your qPCR targets. After P-Q calculates sample dilutions outside of their expected inhibitory ranges, it then calculates the same ng/μL sample per each different target per each final target reaction - all within the valid, non-inhibitory, LOG-linear-amplification-capable, high efficiency range for each target. Therefore, due to dynamically sound, equal sample loading on a per target basis, the use of reference genes becomes theoretically unnecessary; and much of our recent data seems to bear this out. But, a note of caution here: although this is a time-saving and high-throughput idea, it still represents a radical departure from common qPCR practice. Further, in the event that RNA sample spectrophotometer or NanoDrop readings are slightly off (due either to user error or to contaminating genomic DNA contributing to initial RNA sample readings), it is still wise to run one or two appropriate (validated) reference genes and compare final quantitative results with and without figuring them into the calculations. If the same results are apprehended either way, three observations come to light: a) the reference gene(s) are apparently stable; b) the use of reference gene data normalization becomes unnecessary in such cases; c) the P-Q method regarding this point appears to be valid. However, of greatest importance, and vital to allowing qPCR to generate potentially biologically relevant and/or meaningful gene expression data, is the critical need for investigators to require of themselves to use only high quality RNA (assessed for integrity beforehand) exhibiting 260_nm_/280_nm_ purity ratios of 1.8 or greater, before using it in the assay to begin with (1).

### Comprehensive list of PREXCEL-Q Macros

Note: there are many Macro sub-commands buried within the main Macros in P-Q. Virtually every combination of letter and Ctrl key and/or Ctrl-Shift keys has been assigned to a Macro in the creation of this program. But, there are only a handful of these commands that you, the user, need be aware of. Even though the Macros have been explained to some degree already, a comprehensive listing and description of what they each do is surely also helpful, so here is the list:

Ctrl Shift F is the major Starting Macro that is used to globally assimilate all initial user parameter entries into the system within the “Questionnaire.xls” and the “UMES.xls” files. Activate this command from within the “UMES.xls” file when ready. It is usually the very first command you will use for all One-step qPCR modes.

Ctrl Shift D is used to insert optimal parameters for using Invitrogen SuperMix™ Master Mixes (775 nM primers, 150 nM probe and 5.5 mM final [MgSO_4_] or [MgCl_2_] and 7.2 μL sample per 30 μL reaction size). Activate this command from within the “UMES.xls” file. LCM-qPCR mode defaults to this mix.

Ctrl Shift O is used to insert optimal parameters for using BIO-RAD iScript™ Master Mixes (925 nM primers, 150 nM probe and 7.8 μL sample per 30 μL reaction size). Activate this command from within the “UMES.xls” file.

Ctrl Shift E is activated from within the “UMES.xls” file and is used to correct DNase- or RNase-treatment-related errors in the “UMES.xls” file. This command will not correct mistakes in all situations e.g. where a sample or samples are too concentrated to receive ample DNase- or RNase-treatment; in which case(s) you should dilute such samples so their calculated 260_nm_ values are ~0.6 (@ 1:50 dilution). 0.301029996, which = LOG_10_ of 2, is the most reliable reading on a spectrophotometer – as wrought out by Beer’s Law: Absorbance = LOG_10_(I_o_/I_t_) = εcb. Absorbance readings are most accurate when I_t_ is ~50% of I_o_ (e.g. when A = ~0.301), so, ideally, you would like all of your spectrophotometer sample absorbance readings to be as near ~0.301 as possible in all situations. In practice, readings between 0.05 and 1.0 are generally useful as they fall mostly within the linear range of a standard spectrophotometer’s ability to interpret sample transmittance. For P-Q, 260_nm_ samples reading above 0.6 (@ 1:50 dilution) should be diluted so that they would be calculated to read no more than 0.6 (@ 1:50 dilution), however, this is not written in stone and you can ignore this if you wish. Generally, we urge people to dilute samples with readings of 0.8 or higher (@ 1:50 dilution) to ½ strength so they will not trigger error messages in the program (associated with insufficient DNase- or RNase-treatments). The NanoDrop device is exempt from the limitations spelled out above in that it employs an entirely different (LED and fiber-optic-based) technology than most standard spectrophotometers; it is thus, not prone to the same ‘non-linearity’ that spectrophotometers are prone to.

Ctrl x is used to run both Ctrl Shift F, hidden Macro Ctrl Shift J and Ctrl Shift N back-to-back to initiate P-Q into Two-Step qPCR Modes #2 or #4. Activate this command from within the “UMES.xls” file. (Ignore the Ctrl Shift J Macro individually).

Ctrl Shift N is used after Ctrl x to update any Two-Step qPCR-related changes which affect RT and cDNA reactions. This command also auto-finds the required cDNA volumes.

Ctrl y is used to attain non-excessive Master Mix preparation amounts; run at any time, always follow it with Ctrl Shift Z so your parameter adjustments can be assimilated.

Ctrl Shift Z is the Universal System Update Macro which can be run anytime during use of the program. If you feel your parameters have not been assimilated or incorporated into the program, this Macro always updates/assimilates/incorporates all adjustments you have made to the system at any time. (Hidden Macros Ctrl Shift X, Ctrl Shift Q & Ctrl Shift M are all a part of the Ctrl Shift Z Macro).

Ctrl Shift A is used to automatically calculate evenly-spaced Stock I progressive serial dilutions for your Test Plate across the range you specify the upper limit for by the value you enter into cell M28 of the “UMES.xls file.” The M28 value indicates the highest dilution of Stock I solution tested on your Test Plate, and this value also represents your “in-well” dilution of that sample (post-DNase or RNase-treatment but including the dilution specified in “UMES.xls” file cell I13). Activate this command from within the “UMES.xls” file when ready. Follow this always with Ctrl Shift Z. (This Macro relies on region IG11:IT30 of the “UMES.xls” file for its “double-LOG plot” functionality). Notice, in the “zPrintouts.xls” file, within the Test Plate tab (1st sub worksheet tab), that the ideal Ct values you should expect from your chosen Stock I dilution series for your Test Plate are already spelled out in cells D1 through M1. Deviations from these ideal Ct values of course indicate other than 100% efficiency of your qPCR reactions. You can always calculate what any Ct would’ve been had its governing reaction occurred at 100% efficiency by the equation: Ct_observed_ × LOG_2_(E_AMP_) = Ct_@100%Efficiency_ (assuming no inhibition is involved and that you are truly working within the LOG-linear-amplification-capable range of the qPCR assay). Exponential Amplification or E_AMP_ = (E + 1). Efficiency of Amplification or E = [10^(-1/m)^ - 1], where “m” is the slope of a qPCR target’s standard curve when Ct (or CP) is plotted vs. LOG_10_ of: sample dilution factor, template input or target copy number (2). Activate it from within the “UMES.xls” file.

Ctrl Shift C is used to prepare the “TestPlateResultsAnalysis2006.xls” files for use. Activate this Macro only after you have run your Test Plate and have entered your Test Plate Ct values into the appropriate cells in region B197:H208 of the “UMES.xls” file. Activate this Macro from within the “UMES.xls” file. Follow this always with Ctrl Shift Z.

Ctrl Shift B is used during the “Point Selection Process” to incorporate each of the point selections you make (in effort to uncover/reveal the LOG-linear-amplification-capable ranges for each of your targets within the particular Stock I tested) while working within the “TestPlateResultsAnalysis2006.xls” file. Activate this Macro from within the “TestPlateResultsAnalysis2006.xls” file each time your point selections have been changed. Ctrl z is used during the “Point Selection Process” while working within the “TestPlateResultsAnalysis2006b.xls” file to automatically have P-Q select your valid, LOG-linear standard curve ranges based on your entries. Activate this Macro from within the “TestPlateResultsAnalysis2006b.xls” file. Follow this with Ctrl Shift Z.

Ctrl q is used during the “Point Selection Process” while working within the “TestPlateResultsAnalysis2006b.xls” file. Activate this Macro from within the “TestPlateResultsAnalysis2006b.xls” file to incorporate your own standard curve starting and ending dilution choices (which you type into CC8 and CC9 and so on, of the “TestPlateResultsAnalysis2006b.xls” file). Follow this with Ctrl Shift Z.

Ctrl e is used during the “Point Selection Process” while working within the “TestPlateResultsAnalysis2006b.xls” file. Activate this Macro from within the “TestPlateResultsAnalysis2006b.xls” file to incorporate your own standard curve dilution factor choices (which you type into CC10 and so on, of the “TestPlateResultsAnalysis2006b.xls” file). Follow this with Ctrl Shift Z.

Ctrl Shift G uses the “Sample Aiming Device” to automatically calculate the safe dilution of all of your samples to avoid inhibition. But, one “weak” sample here (e.g. a sample with inordinately lower concentration than the others) can ruin it for the rest of the samples here - forcing them to be diluted out further than necessary all on account of one “bum” sample. Activate this Macro from within the “UMES.xls” file when it is prudent to do so. Allow yourself the discipline to refuse the use of “anemic” (low-concentration) samples at times. Re-isolate.

Ctrl Shift W uses the “Sample Aiming Device” to allow you to attain your own desired Tier 1 ng/μL concentration. You would activate this Macro after entering your desired Tier 1 ng/μL value into cell BE14 of the “UMES.xls” file (in the “Sample Aiming Device” region). Activate this Macro from within the “UMES.xls” file when ready.

Ctrl Shift U (is the “Full Strength Sample Option”) which uses the “Sample Aiming Device” to allow you to attain a Tier 1 ng/μL concentration which is equal to your most concentrated standard ng/μL value. Activate this Macro from within the “UMES.xls” file whenever desired (i.e. for LCM-qPCR).

Ctrl w is used to run both Ctrl Shift F and Ctrl Shift H back-to-back to initiate P-Q into LCM One-Step qPCR Mode #5. Activate this command from within the “UMES.xls” file when ready. (In other words, the Ctrl Shift H Macro, by itself, can be ignored entirely - which is analogous to Ctrl Shift J for Two-Step qPCR above).

Ctrl Shift T is used to update/incorporate any changes made to the “LCM Sample Parameter Adjust Region” in cell range L185:N197 during the use of P-Q for LCM-related qPCR (Mode #5). Activate this Macro from within the “UMES.xls” file.

Ctrl i is used to update the “zPrintouts.xls” file at any time the user feels her/his set-up has not been updated for printouts. Ctrl Shift Z always runs the Ctrl i Macro as part of itself, so do not worry about running Ctrl i if you have just run Ctrl Shift Z. Activate this Macro from within any file at any time.

Ctrl m is used to clear the “zPrintouts.xls” file at any time. Activate from within any file.

Ctrl Shift S is used in conjunction with the “Quick Access” function in “UMES.xls” file cell region F64:G70 to quickly attain one’s desired standard curve dilutions by merely selecting the starting dilution and serial factor for up to 7 target standard curves. This Macro automatically adjusts the values in the two ‘TestPlateResultsAnalysis2006.xls’ files to attain your “Quick Access” parameters. Activate this Macro from within the “UMES.xls” file, and always follow it with Ctrl Shift Z.

Ctrl Shift V is used to trim the added residual factor of 0.000000001 off the values incorporated into the ‘TestPlateResultsAnalysis2006.xls’ files as the result of running Ctrl Shift S or Ctrl Shift P. Activate from within “UMES.xls,” follow it with Ctrl Shift Z.

**Figure 3:**
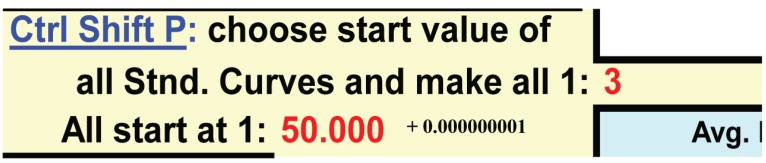


Ctrl Shift P is used in conjunction with cell region F59:H61 near the “Quick Access” region in the “UMES.xls” file that looks like the following (Fig. 3):

To quickly attain the same starting dilution factor and serial progressive dilution factor for all target standard curves, type in your desired starting dilution into “UMES.xls” cell G61 (e.g. “50” in the above picture) and your desired serial progressive dilution factor into “UMES.xls” cell H60 (e.g. “3” in the above picture), then run Ctrl Shift P. Activate this Macro from within the “UMES.xls” file, and always follow it with Ctrl Shift Z.

The Ctrl r Macro resets the “Questionnaire.xls” file, inserts an “m” into “Sample Aiming Device” cell BB29, and inserts “10” into cells F55 and F58 of the “UMES.xls” file. Activate this Macro from within the “UMES.xls” file whenever appropriate. This Macro is already an integral part of the Ctrl Shift K command below.

Ctrl Shift K is the Comprehensive System Reset Macro used to reset the entire P-Q program. It resets all fields to pre-set/surrogate values (of a theoretic 7-target, 72-sample One-Step qPCR set-up). Activate this Macro from within any file when you wish to start from scratch and/or initialize a new instance of P-Q to use for a new qPCR study.

Be sure to activate the exact Macro command(s) you intend: e.g. “Ctrl y” not “Ctrl-Shift y” … be very accurate as to which Macro you activate, and when. In addition, if you unintentionally activate a Macro, hit the Esc key, return to the “UMES.xls” file, and proceed - no damage done (except when accidentally activating the Macro Ctrl a from within files other than the three “MMSetup2006.xls” files. This Macro, if activated accidentally from with inside the wrong file, will require that you start over, unless you have already saved your changes prior to the accidental Ctrl a Macro activation.

### DNase, RT and One-Step vs. Two-Step qPCR Discussion, and Opinions

With DNase treatments ... know your exact conditions, and use them for each and every one of your samples preceding One-Step qPCR. This falls in line with the familiar seed of thought which gives rise to the philosophy that ‘*the more identical your samples are (e.g. make sure that all samples are exposed to identical pre-qPCR preparative methodologies), the more they can truthfully be viewed as things which can actually be compared with and amongst one another in scientifically-sound fashion,*’ etc. But ... for Two-Step qPCR, you see that this ‘*DNase-treatment similarity philosophy*’ is often abandoned in favor of another point of user-attainable sample-*similaritude*: DNase treatments of RNAs destined for RT [cDNA synthesis reactions preceding Two-Step qPCR] are often not uniform at all. But, these DNase-treatments are either preceded or followed by dilutions which allow the same amount of [DNase-treated] RNA to end up in each of the RT [cDNA synthesis] reactions. These pre- or post-DNase treatment dilutions represent the ‘normalising’ feature of Two-Step qPCR that then makes all the samples *similar* at this point - albeit on a basis of sample RNA ng/μL concentration during RT. But, these approaches assume (in addition to all RT reactions containing the same concentration of differentially-diluted RNA) that all RT reactions are not differentially sample-RNA-inhibited (due to each sample being diluted to a different extent in effort to attain the same [ng/μL] for DNase treatment or for RT), and that all RT reactions are formulated correctly so that each RT reaction can be expected to occur at the same RT reaction efficiency [i.e. all reactions converting 2 μg of RNA into 2 μg of cDNA are deemed 100% efficient. Whereas if the reactions converted 2 μg of RNA into 1.6 μg of cDNA the reactions would be assumed to have occurred at 80% RT efficiency, and so on]. Outside the notion: “But reference genes will straighten this whole thing out in the end anyway” ... there may indeed be ‘hell’ to pay here. E.g. what if each different target transcript is reverse transcribed with different efficiency in the presence of differential amounts of inhibitory material per each differently-diluted RNA sample? The differential nature of these reactions depending upon sequence topographies, differential degradation rates of different RNAs and differentially-concentrated inhibitory materials etc. can all collude to affect RT and PCR reactions, and therefore, a user’s ability to get at, and report, the truth. So, for those who perform only Two-Step qPCR, how *does* one establish confidence (outside of sheer RNA sample dilutions which have already been shown to preclude inhibition of RT and/or DNA polymerase enzymes [due to either too much RNA itself or carry-over contaminants from sample preps]) that each RT reaction for each sample indeed goes off at the same (or acceptably similar) RT efficiency in each case - especially when one has not diluted one’s sample RNAs such that they each end up (in-well) diluted (post-DNase treatment) at least 1:50 (for column isolates) or 1:200 (for Trizol isolates) within the final qPCR reactions? Recall that these dilution thresholds (1:50 in-well, 1:200 in-well, etc.) when not heeded, destroy the ability of RT and PCR reactions to work correctly during One-Step qPCR (2). But, why would the RT reactions preceding Two-Step qPCR be exempt? There is one reason which may be good enough: RT enzymes are typically used 5 to 40 times (ABI Multiscribe RT and Invitrogen SuperScript™ III, respectively) more concentrated per unit volume RT reaction preceding Two-Step qPCR than what they are used at for the RT phase of One-Step qPCR. In other words, possibly by sheer attrition, the higher presence of reverse transcriptase enzyme alone may be effective in [overcoming RT-inhibitory factors] providing enough RT activity during RT reactions to maximize the efficiency of RT reactions preceding Two-Step qPCR in most cases; effectively accomplishing the same thing that increased sample dilutions would prior to One-Step qPCR (which uses as little as 0.25 Units/μL RT enzyme for first-strand synthesis in some cases). RT reactions preceding Two-Step may be more resistant to inhibition on account of the higher (Units per μL) presence of RT enzyme than is used (per μL) in many commercial One-Step qPCR reaction formulations. But beware: 10 Units/μL RT enzyme can end up crippling subsequent PCR (qPCR) reactions since RT enzymes (apparently even denatured) can bind cDNA, causing sporadic amplifications during the PCR phase (ABI product literature note regarding use of their Multiscribe™ RT enzyme).

There is also RIBO Green, which investigators can use to quantify cDNAs after each RT reaction to check each RNA sample’s efficiency of transformation into cDNA (25) - but, such analyses could very well invite other misinterpretations - so we have the tendency to avoid the entire debate by sticking with well-rendered One-Step qPCR in as many cases as possible, even though cDNA can last for decades in the freezer. If your RNA samples are extremely precious and/or limited in supply, by all means convert them to cDNA for longevity. But, DNase treat them all identically (same volume of RNA per each DNase reaction - before or after which samples are normalized to the same ng/μL concentration) and subject them each to at least 3.5 to 5 U/μL of a robust RT enzyme for reverse transcription of RNA into cDNA (Invitrogen’s SuperScript™ II or III, Stratagene’s Affinity-Script™, Quanta’s qScript™, Takara’s PrimeScript™ or BIO-RAD’s iScript™) but only after diluting Trizol RNA isolates at least 1:50 (post-DNase treatment) and column RNA isolates at least 1:10 (post-DNase treatment) to attain identical ng/μL concentrations of each RNA preceding reverse transcription to cDNA^b^. Between 10 and 50 ng/μL RNA during RT is normally sufficient. Any more, and rRNA and tRNA (in addition to any unknown inhibitory materials) can begin to impinge on the efficacy of the RT reactions. Following these guidelines allows Two-Step qPCR to reach the precision that well-rendered One-Step qPCR already enjoys.

Differentially tampering with RT reactions (using different amounts of RT enzyme per reaction, etc.) preceding Two-Step qPCR is a game to be avoided entirely, especially when it has been noted by some that only 5 to 10% of the RNA sample is converted to cDNA in their RT reactions. Also, note that random pentadecamers have recently been advertised to be very effective primers to use for RT reactions; cDNA yield is apparently increased by 3-fold and the ability to amplify rare targets later by qPCR is increased by 11-fold (26). Mixtures of poly-(dT) and randomers are also very efficacious and, also, serve to avoid “3-prime bias.”

Truly, a well-rendered Test Plate unravels many mysteries since it shows the user the unique inhibitory characteristic of their samples as a group. Then, once this is known, P-Q calculates the valid ranges of dilution per target for each sample and standard curve, affording the investigator the best chance at generating Ct values that are truly reflective of the relative or absolute abundance of each target of interest.

But, again, for Two-Step qPCR proponents, this requires that you have indeed, unequivocally, already established methods of DNase treatment and cDNA synthesis (via RT) preceding qPCR which allow all of your sample RT reactions to occur at very similar efficiencies. Once this is ‘confidently’ attained, then “reference genes can straighten the rest out” ... that is, if the reference genes *themselves* are reliable! (E.g. Ubiquitin, Ribosomal Protein S15, Elongation Factor-1α, [Bustin, *et al*.]) (10, 25-32). Further, exogenous references or synthetic templates may help light the way out of the ‘unstable endogenous reference gene caveat’ (e.g. non-endogenous, synthetic or nonsense sequences spiked into samples exogenously at a key point during sample preparations can be used as normalisers for each qPCR study) (28). Or, find a way to incorporate the use of expressed ALU repeats and/or STRs for primate qPCR normalization, or satellite sequences for other species, excluding birds and lizards (24).

So, to recapitulate, be clear on your RT reaction formulations: at what dilution of your RNA samples (preceding Two-Step qPCR) are your RNA samples no longer inhibitory to the RT reaction itself? As a rule of thumb, we have found a generally safe range to be 10-50 ng total RNA/μL RT reaction. But, this all depends on how clean the RNA is going into the RT reactions. Or, test the RNA in a One-Step qPCR application (Test Plate) to witness the kinetic landscape of the inhibitory phenomena first-hand, then use this knowledge to properly formulate your RT reactions preceding Two-Step qPCR. E.g. identify the Units of RT enzyme per ng RNA that will work without inhibiting the PCR phase. One should strive to let the ‘*sunlight shine through the reactions*’ - don’t smother your reverse transcription reactions with too much enzyme or too much template.

Many of the above caveats can be avoided by performing One-Step qPCR for all RNA samples after uncovering the valid sample, target and standard curve dilution ranges by Test Plate analyses using P-Q. Wherein all RNAs are DNase-treated identically (same volume of RNA per each DNase treatment reaction [i.e. using TURBO DNase from Ambion/ABI]), then dilute all DNase-treated samples 1:10 with nuclease-free water (with addition of RNaseOUT as part of the 1:10 dilution), then perform qPCR only after having run a Test Plate on a serially-diluted mixture of a portion of each of the samples (“Stock I”) to study the behavior of each qPCR target of interest over an appropriately-informative dilution series in order to identify the valid, non-inhibitory, high-efficiency-of-amplification, LOG-linear-amplification-capable sample dilution ranges for each target. Then, use P-Q to calculate the exact dilutions of all samples and standards and qPCR reaction formulations and to generate the printouts for the entire final set-up (again: all based on the dynamics revealed by the Test Plate). Finally, One-Step qPCR RT/Taq mixes from Invitrogen (SSIII and Platinum Taq-based), Takara (PrimeScript™), BIO-RAD (iScript™) and Stratagene (Affinity-Script™ Brilliant™) are all superb in allowing One-Step qPCR to reach its zenith.

## Footnotes

a The term “in-well,” when applied to the dilution of a nucleic acid sample, refers to how much each sample has incurred post-DNase treatment for One-Step qPCR samples (or post resolubilization if no nuclease treatment is used) and post RT reaction for Two-Step samples by the time the samples actually reside in the final reaction mixture in a well on a reaction plate (or indeed, final reaction tube). The in-well dilution of a sample is thus the combination of the post-DNase (or post RT) dilution of samples (i.e. 1:5 or 1:10 dilution of all samples), the dilution each sample undergoes to attain the ng/μL concentration and non-inhibitory dilution that works for the target which requires the most ng sample per μL reaction, and the dilution each sample incurs merely by being placed into the reaction itself. These are basic things that need to be acknowledged for they too often go unnoticed or unaccounted for. P-Q keeps track of all of this for each sample. [The proportion that sample volumes are per each final qPCR prepared reaction volume is also an essential part of determining the final in-well dilution incurred by each of your samples either post-DNase or RNase treatments (for One-Step qPCR) or from RT reactions on (for Two-Step qPCR). Typically, our samples are 0.24 of each qPCR reaction (e.g. 7.2 μL per each 30 μL qPCR reaction prepared). Yours may differ from that – but, be sure to take this into account when calculating the overall final in-well dilutions of your own samples if not using P-Q].

b [The dilution each RNA incurs (to attain identical ng/μL concentrations) post-DNase but prior to RT, in our experience, should be at least 1:50 for Trizol isolates and at least 1:10 for column RNA isolates, or inhibition of RT reactions prior to the PCR phase of Two-Step qPCR, and the PCR itself, may be reasonably anticipated]. Well-planned qPCR sample dilutions solve many ills – P-Q spells these dilutions out for the user (2). **Miscellaneous note:** Why RNase-treat DNA samples? Differential binding of reverse primer in differentially RNA-contaminated DNA samples. Remember, the hierarchy of nucleic acid affinity, in general, is: RNA:RNA (strongest), RNA:DNA (intermediate) and DNA:DNA (weakest).

## DEDICATION

This effort is dedicated to the ever-present memory of my father, Lyn Marshall Gallup, whose tenacious spirit recreates me daily.

## APPENDIX 1

### Preliminary Important Definitions and Clarifications

Being clear on the following things will help you to use PREXCEL-Q as intended:

**qPCR** refers to “quantitative real-time PCR” which has also been called “QRT-PCR,” “RT-QPCR,” “RT-RT-PCR,” “real-time relative or absolute quantitative fluorogenic PCR,” etc. We use the acronym, “qPCR,” solely to connote this technique.

**One-Step qPCR** refers to any approach to qPCR wherein the original nucleic acid isolate is used directly in a qPCR Master Mix without transformation. This can apply to both RNA and DNA. If your original isolate is RNA, or if your original isolate is DNA, and you use them directly in a qPCR Master Mix (presumably after DNase- or RNase-treating and/or pre-diluting them), you are performing “One-Step qPCR.” It is important to keep the DNase or RNase treatments here identical for every sample. Whether one is DNase-treating RNA or RNase-treating DNA, think of them both as the “same” procedure regarding “template treatment.” Select the appropriate extinction coefficient for the nucleic acid type you are working with in “UMES.xls file” cell range R150:R153. (ε) Extinction coefficient/1000 values are used, where ε = # μg/mL/1 o.d. @ 260_nm_. The initial template type you are entering o.d. 260_nm_ readings for will use an extinction coefficient of: 0.033 for oligo DNA, 0.037 for ssDNA, 0.04 for ssRNA and 0.05 for dsDNA. P-Q keeps track of every dilution all samples (for up to 72 samples in a One-Step qPCR approach when not using the “01AuxSmplFile.xls” file for additional sample entries) incur - from their isolation to their use in qPCR, and is, therefore, always aware of each sample’s ng/μL (DNA or RNA) concentration at every step along the way.

**Two-Step qPCR** refers to preparing cDNA from (usually DNase-treated) RNA first, and then using the pre-prepared cDNA in a qPCR Master Mix. E.g. whenever an investigator intervenes to transform any isolated nucleic acid sample into a complementary nucleic acid in a separate step preceding qPCR, one is performing “Two-Step qPCR.” For such approaches, the user should strive to keep all such nucleic acid ‘transformations’ as identical as possible for all samples (i.e. use the same ng RNA per unit volume reverse transcription [cDNA synthesis] reaction for each sample, etc.); choose one approach for each such manipulation and stick with it for all samples. Use high-efficiency transformations. P-Q keeps track of any and all dilutions all samples (up to 60 samples in a Two-Step qPCR approach when not using the “01AuxSmplFile.xls” file for additional sample entries) incur throughout - from their isolation to their use in qPCR, and is, therefore, always aware of each sample’s ng/μL (cDNA) concentration at every step along the way.

P-Q’s default approach in calculating DNase treatments of RNA preceding its use in reverse transcription (RT) reactions (preceding Two-Step qPCR) breaks away from the constraints mentioned in the above paragraph by allowing one to use different amounts of RNA per unit volume DNase-treatment reaction. This approach allows one to eliminate the addition of water to the DNase reactions (to save on DNase reaction set-up time). One can choose one’s own approach (via the “Questionnaire.xls” file) or use the P-Q default approach for this - both are OK.

It is important to mention the “Questionnaire.xls” file at this point since all new users of this program should fill it out right away. Custom use of P-Q draws on the values and responses placed into this file, so we urge users to fill it out immediately upon opening P-Q. Further, there are similar regions within the “UMES.xls” file that must be answered identically to the responses placed into the “Questionnaire.xls” file. This is described in more detail on page 21 in the section entitled ‘The “Questionnaire.xls” file’ in the current user manual (“PREXCEL-Q Manual 8-1-8”).

## APPENDIX 2

### The ~7 PREXCEL-Q User Modes

After a full reset (Ctrl Shift K) of the system, you have 7 directions to go:

- **Questionnaire.xls file-interfaced One-Step qPCR Mode.** This mode relies upon the values and responses investigators enter into the “Questionnaire.xls” file pertaining to One-Step qPCR and is active when an “x” is entered into cell J7 of the “Questionnaire.xls” file, and a “y” is entered into cell I141 of the “UMES.xls” file. The Ctrl Shift F Macro introduces user values and characters into the system. Updating changes and corrections to set-ups is accomplished by running the Ctrl Shift Z Macro. (For all modes, anytime one changes the number of samples or sample o.d. 260_nm_ information, an initial global Macro must be run again to introduce the new sample parameters into the program. Here, that global Macro would be Ctrl Shift F). The more one enters parameters correctly from the start, the less one will have to re-run various Macros to get things right.
- **Questionnaire.xls file-interfaced Two-Step qPCR Mode.** This mode relies upon the values and responses investigators enter into the “Questionnaire.xls” file pertaining to Two-Step qPCR and is active when an “x” is entered in cell K7 of the “Questionnaire.xls” file and all required identical entries have been made in both the “Questionnaire.xls” and “UMES.xls” files - including the entry of RNA o.d.260_nm_ values into region B120:B191 of the “UMES.xls” file after which the Macro Ctrl x is run. The system automatically enters a “y” entry into cell I141 of the “UMES.xls” file as part of the Ctrl x Macro’s response to the “x” above in K7. Updating changes and corrections to set-ups is accomplished by running the Ctrl Shift Z Macro, but, if changes are made which affect DNase- or RNase-treatment parameters or RT reaction parameters, the Macro Ctrl Shift N should be used instead of Ctrl Shift Z. “zPrinouts.xls” file tab (Excel worksheet) “QuesPQ” provides your sample dilutions preceding DNase (or RNase) and RT reactions, and shows the user the DNase (or RNase) and RT reaction set-ups themselves. The “zPrinouts.xls” “QuesPQ” tab is provided solely for this mode.
- **P-Q default One-Step qPCR Mode.** This mode does not rely upon the values and responses investigators enter into the “Questionnaire.xls” file. It only responds to the values and entries made within the “UMES.xls” file, and is active when an “n” is entered into cell I141 of the “UMES.xls” file. The Ctrl Shift F Macro introduces user values and characters into the system. Updating changes and corrections to set-ups is accomplished by running the Ctrl Shift Z Macro.
- **P-Q default Two-Step qPCR Mode.** When no “x” is entered into cell K7 of the “Questionnaire.xls” file, this mode is active and only responds to the values and entries made within the “UMES.xls” file, and is active when an “n” is entered into cell I141 of the “UMES.xls” file followed by running the Ctrl x Macro. Updating changes and corrections to set-ups is accomplished by running the Ctrl Shift Z Macro, but, if changes are made which affect DNase or RNase-treatment parameters, or RT reaction parameters, Ctrl Shift N should be used instead of Ctrl Shift Z for updating/informing the system of those particular changes. “zPrinouts.xls” file tabs “DNAse2Stp” and “RT2Stp” provide the DNase (or RNase) and RT reaction set-ups.
- **P-Q default LCM One-Step qPCR Mode.** This mode does not rely upon the values and responses investigators enter into the “Questionnaire.xls” file (and therefore does not even require one to type an “x” into cell J8 of the “Questionnaire.xls” file), it only responds to the values and entries made within the “UMES.xls” file and is active when an “n” is entered into cell I141 of the “UMES.xls” file followed by running the Ctrl w Macro (~6 minutes). *Changes to LCM qPCR sample parameters in cell range L185:N197 of the “UMES.xls” file are introduced into the system using the Ctrl Shift T Macro, while updating any other subsequent changes or corrections to set-ups is accomplished by running Ctrl Shift Z. [*Ctrl w initiates P-Q into LCM-qPCR mode].
- **Quick Mode.** This mode does not rely upon the values or responses investigators enter into the “Questionnaire.xls” file, and do not require sample A260_nm_ entries either. It is designed to let users test as many primers, primer-probe sets or [beacon, scorpion, etc.] probes as quickly as possible without regard for sample status or preparation. It doesn’t even respond to most of the values and entries made within the “UMES.xls” file, and is active when an “n” is entered into cell I141 of the “UMES.xls” file and only when non-zero entries are made within “UMES.xls” file cells I187 and I188. Values must also be entered into one or all of cells G190, G191 and G192 to tell the system the nM concentration of primers and/or probe to be used for all assessments. Only one choice for these concentrations is allowed here since it is the non-precise goal of this mode to allow users to merely quickly determine whether or not their primers and/or probe(s) work at all (SYBR Green users can employ this function to test many primer sets quickly). The other requirement for this mode to function is that you must type how many replicates you will use per sample for these assessments in “UMES.xls” cell U25. It is also important that a “1” be typed into “UMES.xls” cell O25 as well. Updating changes to parameters is accomplished by running Ctrl y and Ctrl Shift Z Macros. But, remember, working in this mode stops the functionality of many other aspects of P-Q, and it is important to remember that the output (printout) for this use of P-Q is found only within the “SP MM” tab (spreadsheet) cell range A150:J177 within the “zPrintouts.xls” file. The “zPrintouts.xls” file contains 11 Excel worksheets which house the program’s printouts.
- **Exploratory (Free) Mode.** This is the use of P-Q for other things besides qPCR - any dream you can dream - any other creative use of this program you can think of ...

**P-Q files you will directly use.** During the routine use of this program, you will interact with only 5 (but possibly up to 7) of the 27 P-Q Excel files. These files are:

- “Questionnaire.xls” - for user parameter entries,
- “UMES.xls” - for user parameter entries.
- “TestPlateResultsAnalysis2006.xls” - for analyzing Test Plate Ct values to locate the LOG-linear-amplification-capable, high-efficiency-of-amplification ranges for each qPCR target.
- “TestPlateResultsAnalysis2006b.xls” - also for analyzing Test Plate Ct values to locate the LOG-linear, high-efficiency amplification ranges for each target and to finally select the valid dilution ranges for each of your qPCR target standard curves.
- “zPrintouts.xls” - printouts for all reagent set-ups to use in the lab (11 Excel spreadsheets).
- “01AuxSmplFile.xls” for entering additional sample o.d.260_nm_ readings. (This file is the most recent addition to the program; the 27^th^ file, which allows for more than 60 sample entries for Two-Step and more than 72 sample entries for One-Step qPCR).
- “FinalLabPrintOutforDNaseandmakingcDNAs.xls” - for including the addition of water to DNase or RNase reactions when desired or needed.

Note: The P-Q program relies on users already being familiar with some of the basic mechanics of using a Windows Excel spreadsheet. Users are required to know how to be sure your cursor is not still activated on a cell after you have entered a value or character there, and to know how to ‘Copy’ and ‘Paste Special Values.’ Simply clicking outside a cell after entering a value or character solves skill #1 above, and right-clicking the mouse or looking through the Edit menu in the Excel toolbar guides one to the ‘Copy’ and ‘Paste Special Values’ commands. Using the ‘Copy’ command followed by the ‘Paste Special Values’ command allows one to preserve the original cell formatting in the program and prevents one from inadvertently or unintentionally pasting a formula instead of a value. I urge you to use the ‘Copy’ command followed by the ‘Paste Special Values’ operations in tandem whenever pasting anything into the program’s user-input regions - as opposed to using ‘Copy’ then the ‘Paste’ command. Another thing to note here is that users need not be alarmed by error messages such as #DIV/0!, #VALUE! or #N/A which appear throughout the program in various locations; these are all healthy manifestations of parts of the program that are either not being used (in cases which make the full use of P-Q unnecessary) or, the program is using the error messages to activate formulas in other cells that rely on exactly what kind of error messages they are.
